# Application of the Konno procedure for infective endocarditis in native bicuspid aortic valve with annular abscess extending into the interventricular septum

**DOI:** 10.1093/jscr/rjab428

**Published:** 2021-09-23

**Authors:** Toshio Doi, Kanetsugu Nagao, Hayato Obi, Akihiko Higashida, Masaya Aoki, Shigeki Yokoyama, Saori Nagura, Shigeyuki Yamashita, Akio Yamashita, Kazuaki Fukahara, Naoki Yoshimura

**Affiliations:** First Department of Surgery, University of Toyama, Toyama, Japan; First Department of Surgery, University of Toyama, Toyama, Japan; Department of Cardiovascular Surgery, JA Nagano Koseiren Shinonoi General Hospital, Nagano, Japan; First Department of Surgery, University of Toyama, Toyama, Japan; First Department of Surgery, University of Toyama, Toyama, Japan; First Department of Surgery, University of Toyama, Toyama, Japan; Department of Cardiovascular Surgery, JA Nagano Koseiren Shinonoi General Hospital, Nagano, Japan; First Department of Surgery, University of Toyama, Toyama, Japan; First Department of Surgery, University of Toyama, Toyama, Japan; First Department of Surgery, University of Toyama, Toyama, Japan; First Department of Surgery, University of Toyama, Toyama, Japan

## Abstract

Annular abscess is a serious complication of infective endocarditis, which often requires complex surgery and has a very high post-operative mortality rate. The Konno procedure involves valve annuloplasty for a narrow aortic annulus or left ventricular outflow tract stenosis in children; it is also performed for various cardiac conditions in adults. Here, we report a case of the Konno procedure performed in a patient with aortic valve infective endocarditis, with an annular abscess extending into the interventricular septum (IVS). A 58-year-old man who presented to our hospital with fever was diagnosed with aortic valve infective endocarditis caused by *Streptococcus saccharolyticus*. On echocardiography, an annular abscess in the direction of the IVS was detected, and surgery was planned. The Konno procedure was performed to secure an adequate surgical field and to debride and reconstruct the cavity created by the interventricular septal abscess. The patient was discharged uneventfully 29 days after surgery.

## INTRODUCTION

Annular abscess is a very serious complication of infective endocarditis, which often requires complex surgery and has a very high operative mortality rate of 7.1–36% [1–6]. The Konno procedure has been performed as an aortic annular enlargement technique for narrow aortic annulus and left ventricular outflow tract stenosis in children [7]. There are also several case reports of it being performed in patients with infective endocarditis complicated by an aortic annular abscess [8–11]. In this case, we performed the Konno procedure in a patient with infective endocarditis and with an aortic annular abscess extending into the interventricular septum (IVS).

## CASE REPORT

**Figure 1:**
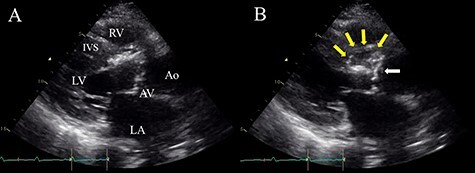
Preoperative TTE (parasternal long-axis view) showing vegetation on the aortic valve (white arrow) and aortic annular abscess extending to the IVS (yellow arrows); (**A**) end-systolic phase; (**B**) end-diastolic phase; LV, left ventricle; RV, right ventricle; LA, left atrium; AV, aortic valve; Ao, ascending aorta.

A 58-year-old man with a history of angina pectoris and diabetes mellitus was admitted to our medical center because of high-grade fever and progressive cough for 4 weeks. On examination, his temperature was 38.5°C, and a grade 3/6 systolic murmur was audible at the right sternal border in the second intercostal space. Laboratory findings showed leukocytosis (white blood cell count (WBC), 18 310 μ/l), high C-reactive protein (CRP) level (10.8 mg/dl) and a high procalcitonin level (3.17 ng/ml). *Streptococcus saccharolyticus* was identified from two samples of blood cultures. Initial transthoracic echocardiography (TTE) revealed severe aortic stenosis, with a mean pressure gradient of 65 mmHg, due to a calcified bicuspid aortic valve and small vegetation attached to the aortic valve. Magnetic resonance imaging of the brain revealed multiple small acute cerebral infarctions. We initially started intensive intravenous antibiotic therapy (sulbactam/ampicillin: 12 g/day, gentamycin: 120 mg/day) based on the diagnosis of infective endocarditis complicated by acute cerebral infarctions. His fever subsided immediately, and the WBC and CRP levels returned to the normal range. However, repeat TTE revealed an aortic annular abscess below the right coronary cusp extending into the IVS (Fig. 1A and B). Therefore, we decided to perform emergent surgery, including aortic valve replacement and radical debridement of the aortic annulus and IVS, on Day 34 after admission.

**Figure 2:**
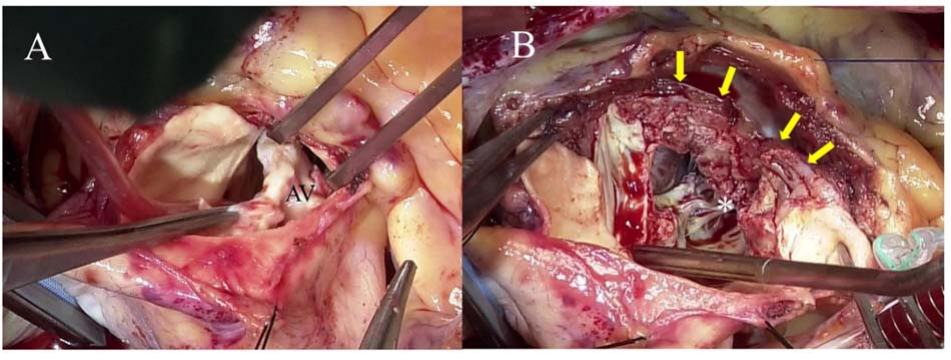
Intraoperative photographs; (**A**) a severely calcified and infected aortic valve was resected; (**B**) aortic annular abscess (asterisk) extending to the IVS (yellow arrow) was opened widely by Konno incision.

**Figure 3:**
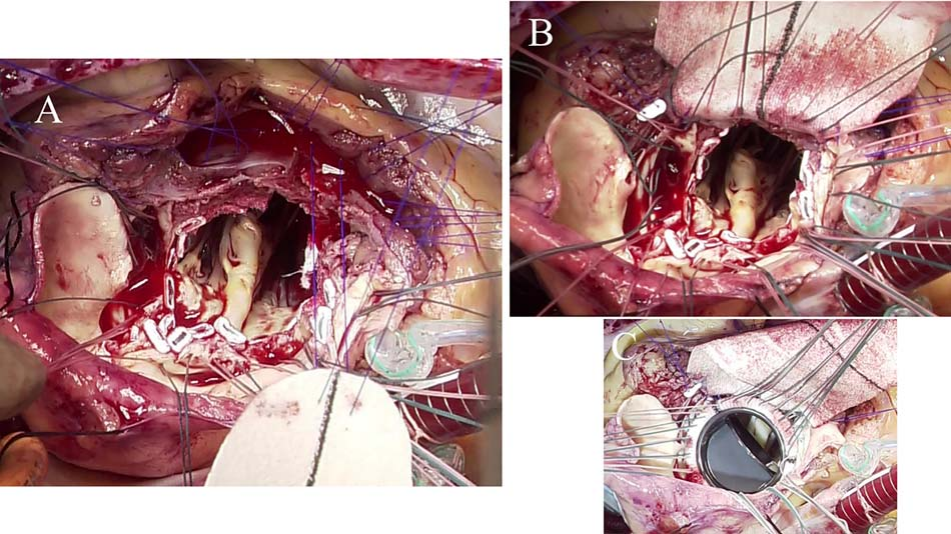
Intraoperative photographs; (**A**) bottom half of the Dacron patch trimmed into the shape of foliage was sutured to the debrided IVS using 4-0 polypropylene mattress sutures with feft-pledgets; (**B**) aortic annulus and IVS were reconstructed by the Dacron patch; (**C**) a St. Jude Medical 27-mm mechanical valve was implanted in the reconstructed aortic annulus.

Following standard median sternotomy, we established cardiopulmonary bypass (CPB) with ascending aortic cannula and drainage from the bicaval cannulae. A left ventricular vent tube was inserted via the right upper pulmonary vein. Under mild systemic hypothermia (30°C), the ascending aorta was clamped and antegrade cold-blood cardioplegia was administered into the aortic root for cardiac arrest. A longitudinal aortotomy was performed at the anterior wall of the ascending aorta toward the left side of the right coronary ostium. The aortic valve was severely calcified; the bicuspid valve was found to be infected and was completely resected (Fig. 2A). An aortic annular abscess under the right coronary cusp and extending into the IVS was detected. The IVS was dissected by the abscess and formed a pouch-like giant cavity. We decided to perform the Konno procedure for extensive debridement of the abscess. Aortotomy was extended beyond the aortic annulus on the left side of the right coronary ostium into the IVS to open the abscess cavity widely, similar to the Konno incision (Fig. 2B). The right ventricular outflow tract was then opened toward the proximal end of the aortotomy, and radical debridement of the abscess was performed (Fig. 2B). The bottom half of the Dacron patch (C.R. Bard, Haverhill, PA), trimmed into the shape of the foliage, was sutured to the incised and debrided IVS using 4-0 polypropylene mattress sutures with felt-pledgets (Fig. 3A and B). A St. Jude Medical 27-mm mechanical valve (St. Jude Medical, St. Paul, MN) was implanted in the reconstructed aortic annulus (Fig. 3C). The top half of the Dacron patch was used to close the aortotomy. The right ventricular outflow tract was closed and enlarged using another Dacron patch using a 4-0 polypropylene continuous suture. Weaning from CPB with a low amount of catecholamines was uneventful. Operation time, CPB time and aortic cross-clamp time were 427, 322 and 262 min, respectively. Tissue cultures of the resected valve and IVS were negative. We continued intravenous antibiotic treatment for 4 weeks after surgery. The patient’s post-operative course was uneventful, except for a complete right bundle branch block, and he was discharged 29 days after surgery. Four years later, the patient was asymptomatic and led a normal daily life. The latest post-operative echocardiography demonstrated normal left ventricular systolic function and no recurrence of endocarditis.

## DISCUSSION

The incidence of valve annular abscess in infective endocarditis is reported to be 9.8–39% [1–6]. It is more common in the aortic valve than in the mitral valve [1–6]. It is a very serious complication that requires complex surgery, and the post-operative mortality rate remains high at 7.3–36% [1–6]. The principle of surgical treatment for infective endocarditis with valve annular abscess is radical debridement of the infected tissue and reconstruction of the defective annulus followed by valve replacement [4–6]. If the lesion is limited, patch reconstruction and valve replacement might be the surgical option, but if the lesion is extensive, more complex surgical techniques, such as aortic root replacement, might be required [4].

If the annular abscess extends further into the surrounding tissue, it might result in an interventricular fistula, pseudoaneurysm or ventricular-aortic discontinuity [1, 4]. David *et al*. reported that 16% of annular abscesses extended into the IVS; however, it is unclear what surgical techniques were used [5]. If the IVS abscess is confined to a shallow area, the surgical field might be adequate for debridement and patch reconstruction. However, if the IVS abscess extends deeply toward the left ventricular apex, radical debridement might be difficult. In such cases, the Konno procedure can be performed to ensure an adequate surgical field and radical debridement.

No difference has been reported in the recurrence rate of post-operative infective endocarditis between prostheses using mechanical or biological valves [4, 6]. The recurrence is thought to be related to the surgeon’s skill to recognize and resect all the infected tissue rather than the type of prosthetic valve used [5, 6]. However, the use of homografts reduces post-operative mortality [12], whereas the availability of homografts is less in Japan. We selected a mechanical valve considering the patient’s age because radical debridement of the infected tissue was possible with the Konno procedure.

Complications of the Konno procedure include prolonged duration of surgery due to the complexity of the procedure and heart failure due to decreased movement of the ventricular septum in the long term. In some cases, an atrioventricular block might occur, and pacemaker implantation might be required. In this case, there was no recurrence of infection at the 4-year follow-up, and echocardiography showed normal left ventricular systolic function; however, careful follow-up is necessary.

## CONCLUSION

We encountered a rare case of aortic valve infective endocarditis with an annular abscess extending into the IVS. The Konno procedure was considered a suitable technique for this condition because it provides a good surgical field and allows radical debridement of the IVS abscess cavity.

## References

[1] Anguera I, Miro JM, Evangelista A, Cabell CH, San Roman JA, Vilacosta I, et al. Aorto-cavitary fistula in endocarditis working group. Periannular complications in infective endocarditis involving native aortic valves. Am J Cardiol 2006;98:1254–60. 17056342 10.1016/j.amjcard.2006.06.016

[2] Graupner C, Vilacosta I, SanRomán J, Ronderos R, Sarriá C, Fernández C, et al. Periannular extension of infective endocarditis. J Am Coll Cardiol 2002;39:1204–11. 11923047 10.1016/s0735-1097(02)01747-3

[3] Anguera I, Miro JM, Cabell CH, Abrutyn E, Fowler VG Jr, Hoen B, et al. ICE-MD investigators. Clinical characteristics and outcome of aortic endocarditis with periannular abscess in the International Collaboration on Endocarditis Merged Database. Am J Cardiol 2005;96:976–81. 16188527 10.1016/j.amjcard.2005.05.056

[4] Baumgartner FJ, Omari BO, Robertson JM, Nelson RJ, Pandya A, Pandya A, et al. Annular abscesses in surgical endocarditis: anatomic, clinical, and operative features. Ann Thorac Surg 2000;70:442–7. 10969660 10.1016/s0003-4975(00)01363-1

[5] David TE, Regesta T, Gavra G, Armstrong S, Maganti MD. Surgical treatment of paravalvular abscess: long-term results. Eur J Cardiothorac Surg 2007;31:43–8. 17140802 10.1016/j.ejcts.2006.10.036

[6] David TE, Gavra G, Feindel CM, Regesta T, Armstrong S, Maganti MD. Surgical treatment of active infective endocarditis: a continued challenge. J Thorac Cardiovasc Surg 2007;133:144–9. 17198801 10.1016/j.jtcvs.2006.08.060

[7] Konno S, Imai Y, Iida Y, Nakajima M, Tatsuno K. A new method for prosthetic valve replacement in congenital aortic stenosis associated with hypoplasia of the aortic valve ring. J Thorac Cardiovasc Surg 1975;70:909–17. 127094

[8] Goh K, Yamamoto H, Tsuda N, Sasajima T. Konno procedure for infective endocarditis involving aortic valve in a small child. Ann Thorac Surg 2000;69:1264–6. 10800838 10.1016/s0003-4975(99)01453-8

[9] Black MD, Walley VM, Keon WJ. Fibrous skeleton endocarditis: repair using Konno procedure. Ann Thorac Surg 1994;57:225–8. 8279901 10.1016/0003-4975(94)90408-1

[10] Mavroudis C, Wampler J, Hodsden JE, Rees AH, Solinger RE, Elbl F. Modified aortoseptoplasty for anular abscess and erosion of the membranous septum. Chest 1984;85:442–4. 6697804 10.1378/chest.85.3.442

[11] Vermeulen F, Swinkels B, van Boven WJ. Konno procedure for prosthetic aortic valve endocarditis: 34-year follow-up. Ann Thorac Surg 2012;93:302–4. 22186453 10.1016/j.athoracsur.2011.06.070

[12] Knosalla C, Weng Y, Yankah AC, Siniawski H, Hofmeister J, Hammerschmidt R, et al. Surgical treatment of active infective aortic valve endocarditis with associated periannular abscess-11 year results. Eur Heart J 2000;21:490–7. 10681490 10.1053/euhj.1999.1877

